# Left Common Carotid Artery Agenesis With Independent Origins of the External and Internal Carotid Arteries From the Aortic Arch: A Rare Case in a Patient With Ankylosing Spondylitis

**DOI:** 10.7759/cureus.28456

**Published:** 2022-08-26

**Authors:** Ahmed M AlAni, Ahmad N Al-Ekeer, Jouhar J Kolleri, Abdalla Omer Maki

**Affiliations:** 1 Radiology Department, Hamad General Hospital, Doha, QAT; 2 Clinical Imaging Department, Hamad Medical Corporation, Doha, QAT

**Keywords:** ankylosing spondilytis, aortic arch, magnetic resonance angiography (mra), left common carotid artery agenesis, rare anatomical variant, ct angiogram

## Abstract

The agenesis of the left common carotid artery, with independent origins of the external and internal carotid arteries from the aortic arch, is a well-described but extremely rare congenital anomaly. We present a case of agenesis of the left common carotid artery with the independent origin of the left internal and left external arteries from the arch of the aorta in a patient with ankylosing spondylitis which was depicted by CT angiogram and magnetic resonance angiography. The diagnosis of this anatomical variant especially before interventional procedures is of great importance, as it could complicate the catheterization of these arteries.

## Introduction

Arterial congenital anomalies are progressively being identified in adult patients owing to the widespread use of color-coded duplex ultrasonography, computed tomography angiography (CTA), and magnetic resonance angiography (MRA) for the evaluation of major intracranial and cervical arteries in clinical practice.

Imaging of the aortic arch and brachiocephalic vasculature is routinely performed as part of any investigation of the etiology of neurologic events. Considering the accessibility and feasibility of these advanced radiographic modalities, multiple anatomic variations that may or may not contribute to symptomatology are being characterized [1]. The interpretation of various congenital vascular anomalies can be challenging in diagnostic, therapeutic, and clinical practice, due to the scarcity of similar cases [2]. 

## Case presentation

**Figure 1:**
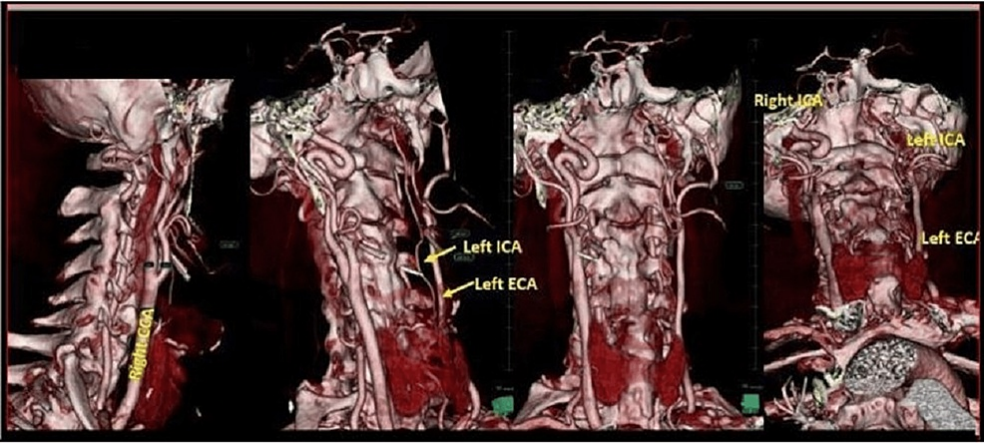
CT angiography 3D reformatting technique shows a normal anatomy of the right CCA, right ECA, and right ICA. Small size left ICA and left ECA originated directly from the aortic arch. CCA, common carotid artery; ECA, external carotid artery; ICA, internal carotid artery.

**Figure 2:**
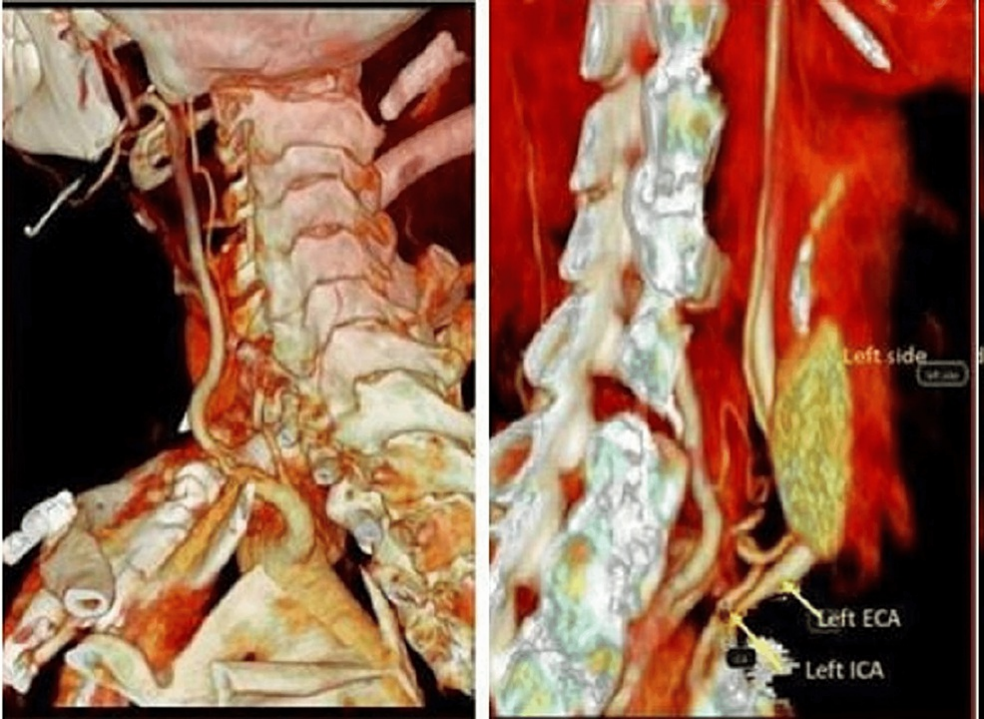
CT angiography 3D reformatting technique shows independent origins of the left ECA, and the small size left ICA from the aortic arch. ECA, external carotid artery; ICA, internal carotid artery.

**Figure 3:**
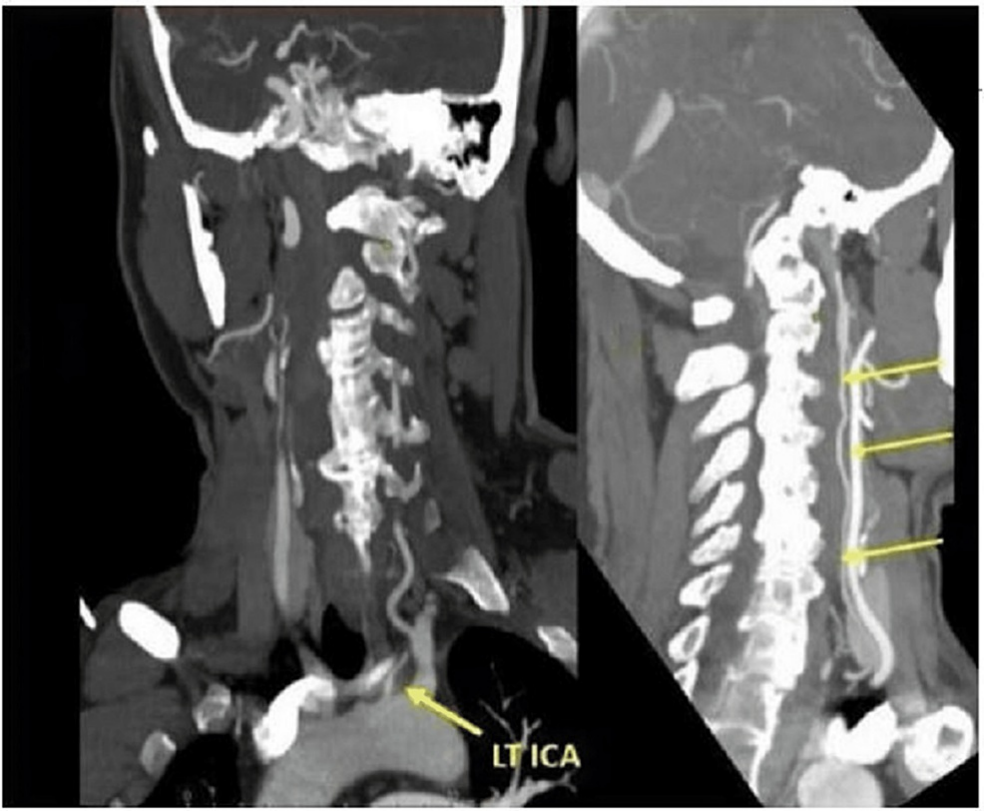
CT angiography with maximum intensity projection shows the small independent origin of the Lt ICA from the aortic arch. Lt ICA, left internal carotid artery.

A 35-year-old male patient, a known case of ankylosing spondylitis (AS), presented with sudden-onset left-sided numbness, weakness, slurred speech, and dizziness. This was the first time to have such symptoms. Symptoms lasted for 15 minutes, then the patient improved and returned to the baseline. Clinical examination revealed normal tone, power, refluxes, no sensory deficit, and normal gait. CT head was performed and was unremarkable. He also underwent CT angiography, which revealed normal right common carotid and both right external and internal carotid arteries, absence of left common carotid artery, left internal and external carotid arteries that are separately originating from the aortic arch, and small attenuated left internal carotid artery (ICA) with dominant supply from the right side (Figures 1, 2, 3).

**Figure 4:**
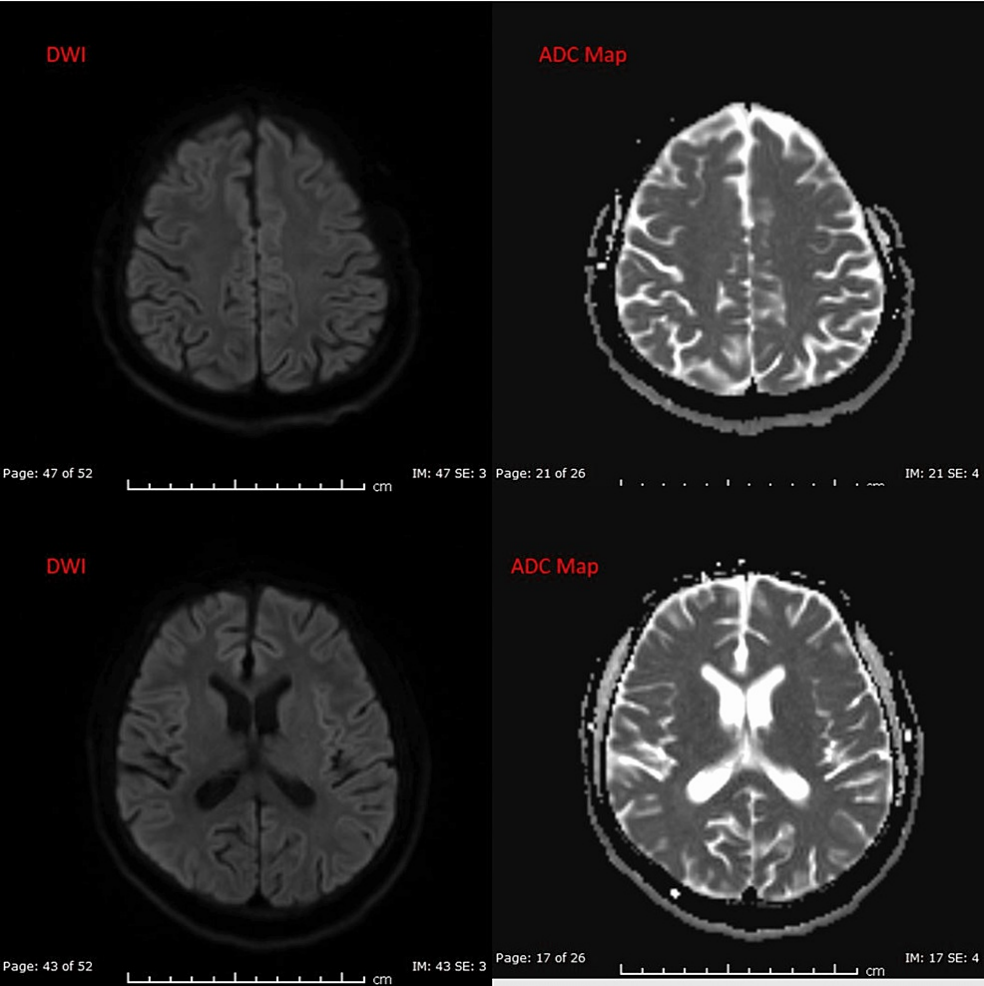
MRI brain, DWI sequence and ADC map showing no focal parenchymal areas of diffusion restriction. DWI, diffusion-weighted images; ADC, apparent diffusion coefficient.

**Figure 5:**
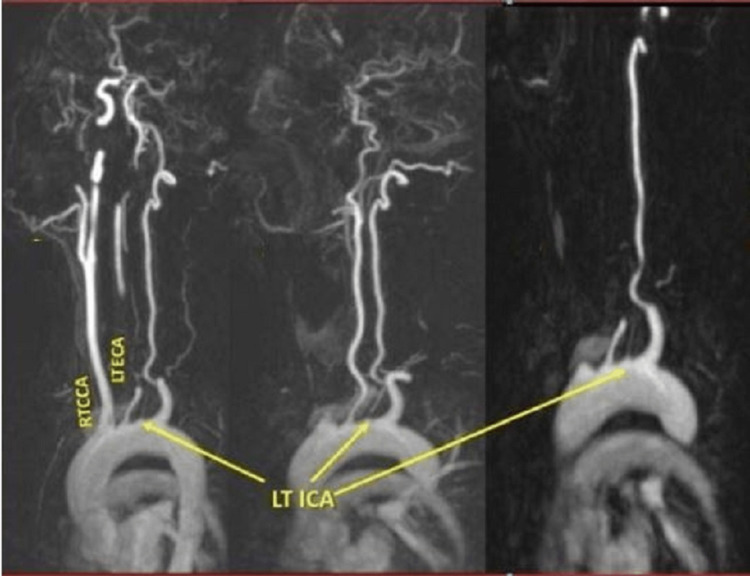
Magnetic resonance angiography shows independent origins of the left ECA and the left ICA from the aortic arch. RTCCA, right common carotid artery; LTECA, left external carotid artery; LTICA, left internal carotid artery.

**Figure 6:**
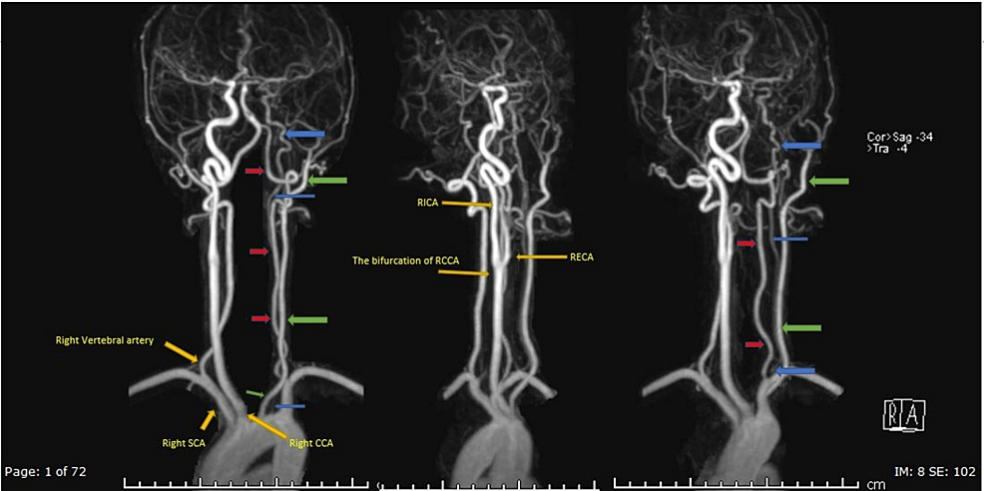
Contrast-enhanced MR angiography with three-dimensional MR digital subtraction technique; three images show the left internal carotid artery (blue arrows), left external carotid artery (green arrows), and left vertebral artery (red arrows). The right CCA, the bifurcation of right CCA, the RICA, the RECA, the right SCA, and the right vertebral artery are separately demonstrated by yellow arrows. CCA, common carotid artery; RICA, right internal carotid artery; RECA, right external carotid artery; SCA, subclavian artery.

**Figure 7:**
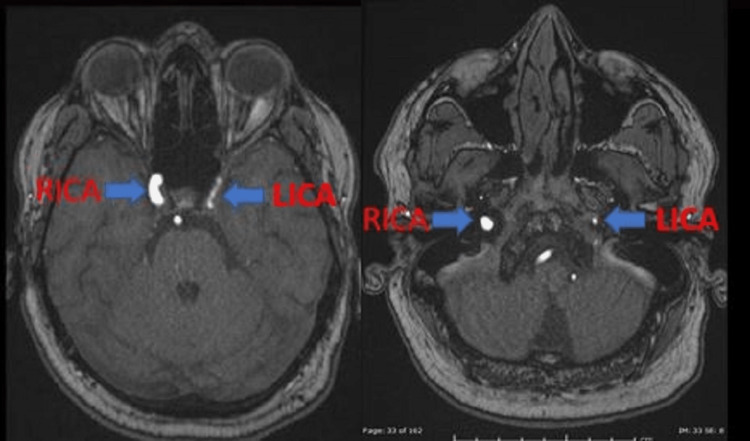
Brain magnetic resonance angiography shows a small LICA compared to the RICA as indicated by blue arrows. LICA, left internal carotid artery; RICA, right internal carotid artery.

**Figure 8:**
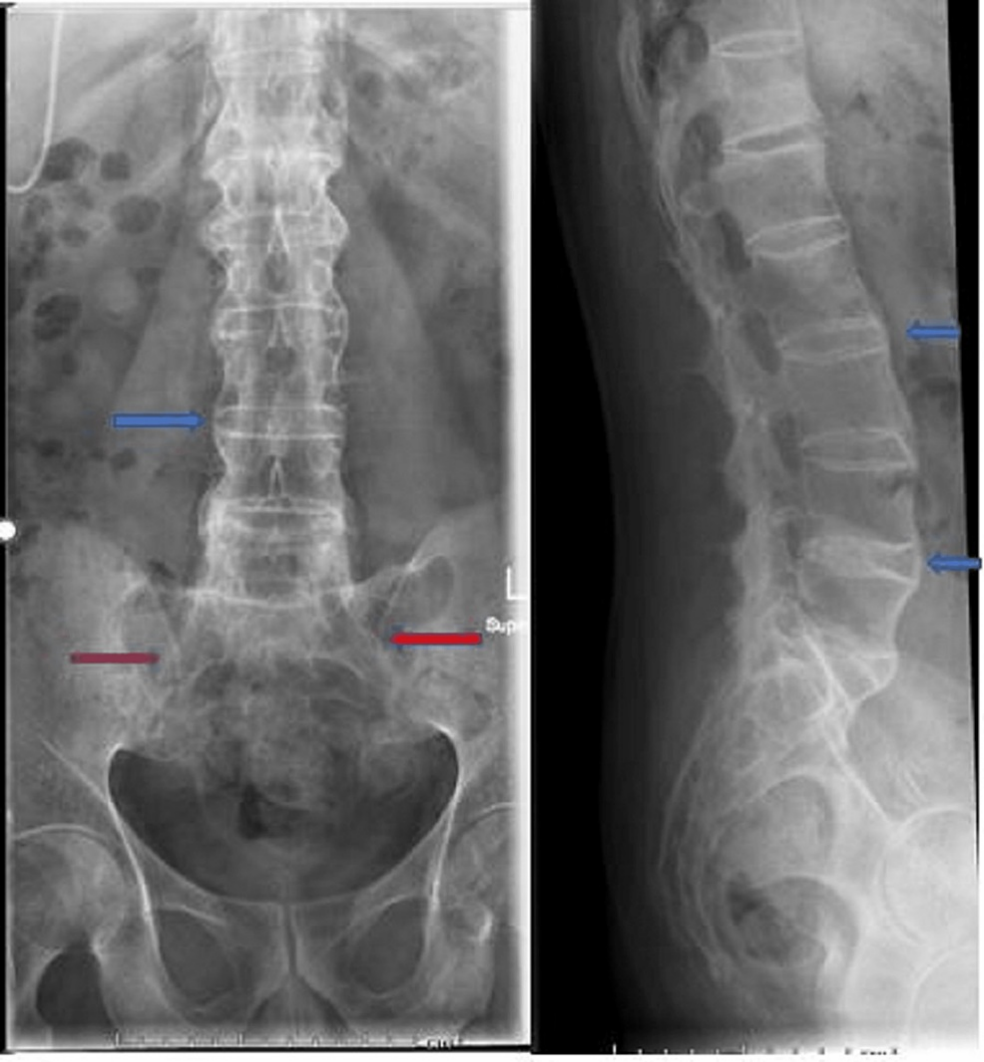
X-ray lumbar spine and sacroiliac joints show ankylosed sacroiliac joints (red arrows), ossification of the annulus fibrosis and fusion of the lumbar spine (blue arrows) giving the typical shape of a bamboo spine.

During the hospital course, the patient underwent magnetic resonance imaging (MRI) which appeared normal with no focal parenchymal areas of diffusion restriction in the diffusion-weighted images sequence (Figure 4). MRA is also done, which delineated the vascular anatomy more clearly, as it revealed diffuse reduced caliber of the left ICA, and the left external and internal carotid arteries arising from the aortic arch separately. The absence of the right brachiocephalic artery is also demonstrated, and the right common carotid artery and right subclavian artery arising directly from the arch, then five arterial branches arising from the aortic arch are noticed. The right ICA, the right external carotid artery as well as the bilateral vertebral arteries appeared unremarkable (Figures 5, 6, 7). In the hospital the patient complained of bilateral ankle pain which limited his ambulation; given the history of AS, a rheumatology consultation was done and tendonitis was considered, and the patient was started on non-steroidal anti-inflammatory drugs (NSAIDs; celecoxib), and he also underwent X-ray lumbar spine and sacroiliac joint for confirmation and documentation. Previously no images were available in records. X-rays show typical features of AS in the form of ossification of the annulus fibrosis and fusion of the lumbar spine as well as ankylosed sacroiliac joints (Figure 8).

Stroke workup including echocardiography and Holter monitoring was unremarkable. Thrombophilia and vasculitis workup including antineutrophil cytoplasmic antibody (ANCA), antinuclear antibody (ANA), anticardiolipin, homocysteine, factor V, factor II, protein S, and protein C were also done and returned negative. The patient blood pressure value was 136/90, glycated hemoglobin (HbA1c) was 6.1, and low-density lipoprotein (LDL) was 3.5. The patient was diagnosed with a transient ischemic attack (TIA), started on aspirin and statin treatment, and discharged home with neurology stroke and rheumatology clinic follow-up. Upon discharge, the patient has returned to his normal state and feels fully improved.

## Discussion

Malacarne published the first case report of agenesis of the common carotid artery in 1784 and its embryologic development and angiographic characteristics were characterized by Lie [3,4]. Supsupin was the first to demonstrate the bilateral absence of the common carotid artery on a CT angiogram [5]. There are five cases of agenesis of bilateral common carotid artery reported in the literature; two of them were associated with symptomatic stenosis and three with intracranial aneurysms [6]. 

Several theories have been proposed describing the origin of these anomalies in terms of the classical conception of aortic arch differentiation. In an embryo, the ductus caroticus involutes, and the common carotid artery is formed between the third and fourth arches from the root of the ventral aorta. The third aortic arch persists as the proximal portion of the ICA. If both the third arches persist and both the fourth arches involute with a high cervical aortic arch, the ECA and ICA would have their own separate origin. The other theory proposes that the third arch is obliterated while the ductus caroticus remains intact, resulting in independent ICA and ECA origins [7].

Boyd gave another explanation for the absent or short common carotid artery [8]. According to him, the ECA may arise from the third arch vessel near the aortic sac, which makes the common carotid artery short, and if it originates farther caudally, it might result in the absence of a common carotid artery. Basilar non-union is a more frequent angiographic finding that results from the failure of longitudinal neural artery fusion and the regression of the bridging arteries that connect the longitudinal arteries and is often associated with the formation of the aneurysm [9].

Anatomically the aortic arch lies between the ascending and the descending aorta, starts when the aorta exits from the pericardium, then goes up diagonally to the left anterior to the anterior part of the trachea, then descends to the left of the fourth thoracic vertebral body, and at this point continue to be descending aorta [10]. 

The aortic arch has a concave shape, with normally three arterial trunks arising anterior-posteriorly from the superior convex part: the brachiocephalic (innominate) artery, the left common carotid artery, and the left subclavian artery. The origins of these arteries are crossed anteriorly by the brachiocephalic vein. The brachiocephalic artery measures about 5 cm in length; it originates beneath the mid-portion of the manubrium of the sternum, after that goes up obliquely to the right then at the level of the right sternoclavicular joint gives the two terminal branches, the right subclavian artery and the right common carotid artery [10]. Then, the left common carotid and left subclavian arteries arise separately from the aortic arch and ascend in a semi-spiral pattern to reach the left side of the neck [10]. 

According to the retrospective study conducted by Karacan et al. to analyze the frequency of variation of aortic arch branching for a total of 1000 patients with a normal left-sided aortic arch who underwent computed tomographic angiography for different reasons, none of the patients were found to have agenesis of the common carotid artery with the internal and external carotid arteries arising separately from the aortic arch on one or both sides [11]. 

In our case, the patient has agenesis of the left common carotid artery, and the left internal and external carotid arteries arise independently from the aortic arch, which is extremely rare. The gold standard modality for diagnosing carotid artery diseases is digital subtraction angiography [12]. However, MRA has a high resolution and diagnostic accuracy, and other used modalities include color Doppler sonography and CTA [13]. 

The absence of a common carotid artery is usually asymptomatic unless it is associated with an arterial lesion. In our case, the patient has symptoms of left-side weakness and numbness which is likely due to TIA. Our patient has also AS. However, at least until now, there is no evidence of a correlation between AS and this rare normal variant in the literature and needs further research. 

## Conclusions

We reported a rare case of agenesis of the left common carotid artery with separate origin of left internal and external carotid arteries from the aortic arch in a patient with AS who presented as a case of TIA. The awareness of this variant is helpful for radiologists and physicians in image interpretations and interventional procedures. 
